# Passive smoking in babies: The BIBE study (Brief Intervention in babies. Effectiveness)

**DOI:** 10.1186/1471-2458-10-772

**Published:** 2010-12-20

**Authors:** Guadalupe Ortega, Cristina Castellà, Carlos Martín-Cantera, Jose L Ballvé, Estela Díaz, Marc Saez, Juan Lozano, Lourdes Rofes, Concepció Morera, Antònia Barceló, Carmen Cabezas, Jose A Pascual, Raúl Pérez-Ortuño, Esteve Saltó, Araceli Valverde, Mireia Jané

**Affiliations:** 1 Fundació Atenció Primaria, Department of Health, Generalitat de Catalunya, Barcelona, Spain; 2 ABS Florida Nord, L'Hospitalet de Llobregat, Spain; 3 ABS Barcelona, Pg. St. Joan, Barcelona, Spain; 4 Department of Medicine, Universitat Autònoma de Barcelona, Barcelona, Spain; 5 Grupo Cardiocat de la Red REDIAPP, Barcelona, Spain; 6 USR Barcelona, IDIAP Jordi Gol, Barcelona, Spain; 7 ABS Montgat, Barcelona, Spain; 8 Grup de Recerca en Estadística, Economía Aplicada i Salut (GRECS), Universitat de Girona, Girona, Spain; 9 CIBER de Epidemiología y Salud Pública (CIBERESP), Spain; 10 Hospital Universitari de Sant Joan de Reus, Reus, Spain; 11 Àrea d'Avaluació. Direcció d'Atenció Primària. ICS. Girona, Spain; 12 Direcció General de Salut Pública. Department of Health, Generalitat de Catalunya, Barcelona, Spain; 13 Grup de Recerca en Bioanàlisi i Serveis analítics, IMIM, Barcelona, Spain; 14 BIBE study group, Barcelona, Spain

## Abstract

**Background:**

There is evidence that exposure to passive smoking in general, and in babies in particular, is an important cause of morbimortality. Passive smoking is related to an increased risk of pediatric diseases such as sudden death syndrome, acute respiratory diseases, worsening of asthma, acute-chronic middle ear disease and slowing of lung growth.

The objective of this article is to describe the BIBE study protocol. The BIBE study aims to determine the effectiveness of a brief intervention within the context of Primary Care, directed to mothers and fathers that smoke, in order to reduce the exposure of babies to passive smoking (ETS).

**Methods/Design:**

Cluster randomized field trial (control and intervention group), multicentric and open. Subject: Fathers and/or mothers who are smokers and their babies (under 18 months) that attend pediatric services in Primary Care in Catalonia.

The measurements will be taken at three points in time, in each of the fathers and/or mothers who respond to a questionnaire regarding their baby's clinical background and characteristics of the baby's exposure, together with variables related to the parents' tobacco consumption. A hair sample of the baby will be taken at the beginning of the study and at six months after the initial visit (biological determination of nicotine). The intervention group will apply a brief intervention in passive smoking after specific training and the control group will apply the habitual care.

**Discussion:**

Exposure to ETS is an avoidable factor related to infant morbimortality. Interventions to reduce exposure to ETS in babies are potentially beneficial for their health.

The BIBE study evaluates an intervention to reduce exposure to ETS that takes advantage of pediatric visits. Interventions in the form of advice, conducted by pediatric professionals, are an excellent opportunity for prevention and protection of infants against the harmful effects of ETS.

**Trial Registration:**

Clinical Trials.gov Identifier: NCT00788996.

## Background

Environmental tobacco smoke (ETS) consists of a mixture of smoke produced by the burning of cigarettes and the smoke directly exhaled by smokers. A large part of the smoke that is inhaled by a passive smoker is made by the burning of cigarettes, which contains certain toxic components at levels far above those inhaled by the actual smoker. It has been shown that the levels of nicotine and tar produced by burning cigarettes are three times higher than in the smoke directly exhaled by smokers, and the concentration of carbon monoxide (CO) approximately five times higher [1]. In addition, the spontaneous burning of cigarettes produces smaller particles that are able to penetrate deeper areas of the lungs [2].

Since the milestone publication by Doll and Hill in 1950, it has been known that tobacco is harmful to health [3], but until 1974 no one had talked about smoking in fathers/mothers, ETS and the consequences for children. Currently, we have evidence [4] that the smoking in parents is associated with various adverse effects on infant health: increased risk of sudden infant death syndrome [5], acute respiratory diseases, respiratory symptoms, aggravation of symptoms in patients with asthma, acute and chronic diseases in the middle ear and slowed lung growth [6,7]. Passive smoking is the leading cause of preventable death in infancy in industrialized countries (third cause of preventable death in adults). According to the International Agency for the Research on Cancer (IARC) of the World Health Organization (WHO), second hand smoke is a type A carcinogen [8]. Furthermore, the WHO MPOWER report [9] proposes suggestive evidence between environmental exposure to tobacco smoke and the following diseases: leukemia, lymphoma, brain tumors and asthma. It is particularly worrying in younger children because they are dependent on adults and therefore cannot avoid exposure to tobacco smoke; moreover, their immune systems are underdeveloped.

In Spain, the law on tobacco control measures (28/2005) has led to notable progress in reducing ETS in work places and in increasing the awareness of the damage caused by ETS [10]; however, at the same time this law has made the home one of the few places where people can smoke [11]. As an example of the exceptions and contradictions of the Spanish law, in "small" places of entertainment (less than 100 square meters) such as bars and restaurants, the owners can decide whether or not to apply a smoke-free policy. Preliminary estimates indicate that 90-95% of small establishments have opted to allow smoking, furthermore, the Spanish law permits the access of children who go in the company of their parents [12]. In Catalonia, various interventions have been carried out to reinforce smoking cessation during pregnancy [13,14]. ETS has the greatest effect on the health of children under the age of five, given that this group of individuals tends to spend the most time at home, making them the most vulnerable to the effects of passive smoking [15-17]. There are few studies on measures to reduce ETS in the home [18]. Various studies have shown that the potential for tobacco pollution at home is more significant than levels of atmospheric contamination. Nonetheless, up to 75% of mothers that smoke expose their newborns to smoke, and between 47-60% of their babies present significant levels of urinary cotinine [19,20].

Interventions to reduce passive smoking have obtained irregular results [21-26]. The Cochrane review [27] on the subject states that there is no sufficient evidence to support any one intervention's effectiveness in reducing smoking habit in parents and the exposure in children. Various studies have established that the level of nicotine in hair is a good quantitative indicator to measure the environmental exposure of tobacco smoke [28-32].

Health education messages from pediatric professionals are an excellent opportunity and, in general, are well accepted by parents since they make reference to the health of their son or daughter. Brief interventions, according to the context and impact of the content, can be just as effective as more intensive interventions [33]. Studies about this type of intervention have not been published in our country.

It is important to conduct methodologically sound studies with a more appropriate design (especially in the calculation of the sample size and monitoring) and evaluation through objective measure of results, such as the level of nicotine in hair.

### Objectives

The principle objective of the BIBE study is to determine the effectiveness of a brief intervention directed at fathers and/or mothers that smoke in order to reduce ETS in babies, in the context of pediatric visits in primary care.

The secondary objectives of the BIBE study are to determine if this intervention produces changes in the tobacco consumption of the parents and subsequently, to study the concordance between the subjective measures of ETS and the objective measures (questionnaire versus biological analysis of nicotine in hair).

The specific objective of this article is to describe the protocol of the BIBE study.

## Methods/Design

### Study design

Multi-centric, open cluster randomized field trial. Unit of randomization: Primary Care teams formed by a pediatrician and a pediatric nurse, both responsible for the same infant population.

### Setting

Pediatric Primary Care consultations in Catalonia. All of the basic health areas of Catalonia will be offered participation through the *xarxa de centres sense fum *(network of smoke free centers).

### Study period

The preparation and pilot test period: during 2008

Data collection period: March-December 2009. The period is not coincidental; the first recruitment visit is in spring and the six-month control visit in the fall, in order to compare the degree of exposure in a similar climate period.

### Study population

**Figure 1:**
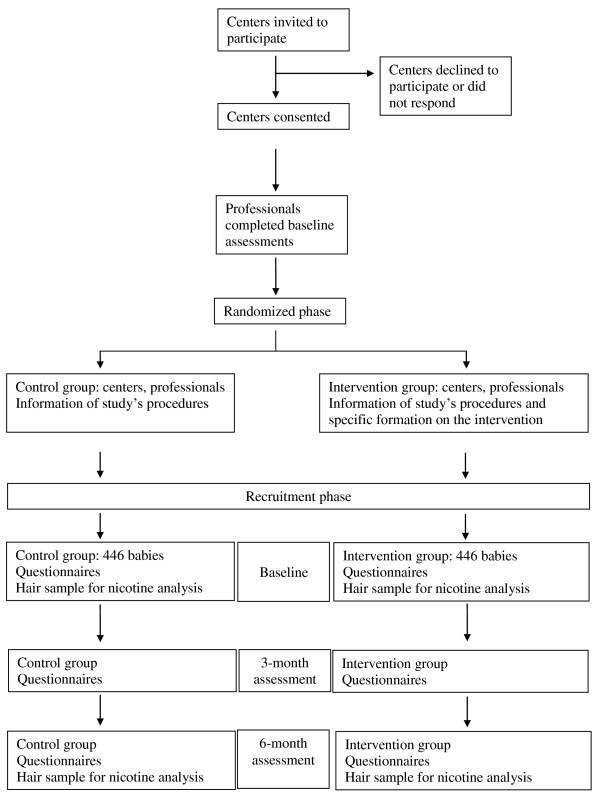
**Flow diagram of the BIBE study**. Diagram and flow chart of the BIBE study.

The study subjects are babies under 18 months of age at the time of recruitment, with a mother and/or father that is an active smoker and that attend a pediatric consultation (with the doctor or nurse) in Primary Care for a check-up visit. Figure 1 shows the diagram flow of the study.

### Sampling strategy

During the two-month recruitment period, and until obtaining the eight proposed cases in each team, all of the mothers and/or fathers that visit with a baby under the age of 18 months will be asked whether or not they are smokers.

### Inclusion criteria

Babies under 18 months of age whose parents answer affirmatively to the question "Do you or your partner smoke?" and who give their informed consent to participate in the study.

### Exclusion criteria

- Any pathology or condition that makes inclusion/follow-up difficult at three and six-months (at the professional's criteria), for example parent or baby with an active, acute disease, known addiction in the parents to other substances or foreseeable change in residency.

- Parent in smoking cessation process at the recruitment phase of the study.

- Refusal of parents to participate in the study.

### Sample size

Based on the data from the pilot study in 46 babies and using the same analysis technique of nicotine in hair used in the present study, a mean of 6.83 ng/mg and a standard deviation of 8.88 ng/mg have been obtained. Assuming a mean difference of 30% between the intervention group and the control group in nicotine in hair at the end of the intervention, with an alpha value of 0.05 and a power of 80%, 295 subjects are needed in each group. If we assume a loss of 20%, the sample size increases to 708 (354 in each group). It is considered feasible for each basic care unit to recruit eight babies in a period of two months. The estimates of the intra-cluster correlation coefficient in cluster randomized trials that evaluate the implementation of clinical practice guidelines using outcome variables in primary healthcare are generally less than 0.05 [34]. These intra-cluster correlation coefficients translate to a cluster size of eight in a design effect corresponding to a factor of 1.4. Therefore, the final simple size will be 992 babies (446 in each group).

### Data collection procedures

There will be three visits with each baby included in the study in both, control and intervention group: a visit at the start of the study (recruitment) and two follow-up visits, at three and six months after the recruitment visit (Figure 1).

The father and/or mother will be asked to provide, using an electronic data collection system created for the study, data regarding the following:

1-The baby: age, sex, breastfed/artificial milk, living nucleus, medical history and clinical data for follow-up,

2- The parents: age, sex, socio-demographic data, smoking status (yes or no). In smokers, data on the smoking habit, smoking behavior during pregnancy and whether or not the baby was breastfed, behavior during breastfeeding. Finally, smoking behavior changes during the study.

3-The degree of exposure of the baby to ETS, within and outside of the home, will be based on the questions proposed by clinical practice guidelines [13,14,35,36].

In order to measure the levels of biological nicotine in hair, a sample of the baby's hair will be taken at the first (recruitment) visit and at the last (six-month) visit. A lock of hair will be cut from the root and once collected, will be placed on a card and put into an envelope, which will be provided with the study materials (Additional files 1 and 2).

For the secondary objective, change in smoking habit in the father and/or mother, expired carbon monoxide will be measured in those that declare smoking abstinence during the study period. Cut off point: 10 ppm.

### Description of laboratory analysis technique

Measuring nicotine in hair provides better information about average or long-term exposure to ETS as compared to other biological markers such as cotinine in the urine, saliva or blood, which have a shorter retrospectivity [28-32,37]. As hair grows in average at a rate of one centimetre per month, nicotine concentrations found in a segment of hair are related to the exposure during a period of time depending on the distance from the root. It is a specific biological indicator, sensitive, stable over time and temperature, not influenced by specific exposures prior to the analysis and easy to obtain [38,39]. The same IMIM team, with broad experience with the technique, analyzed all the samples. The analytical procedure of nicotine in hair utilizes ultra performance liquid chromatography (UPLC) coupled to tandem mass spectrometry (MS/MS) using a triple quadrupole instrument. The hair samples (several milligrams) are extensively washed with dichloromethane to eliminate any superficial contamination. Then hair samples are dissolved in an alkaline (1 M potassium hydroxide) solution. The nicotine released from the matrix of hair is extracted using dichloromethane and an aliquot directly analyzed chromatographically using an HILIC column. Detection by mass spectrometry: monitoring the transition between the pseudo molecular ion ([M+H]+ a m/z 163) and the fragment at m/z 117. The procedure uses a deuterated internal pattern D4-nicotine, which monitors the equal transition (m/z 167-- > 121). The established limit of quantification is 0.5 ng (nanogram) of nicotine in the sample (e.g. 0.05 ng/mg in 10 mg simple).

### Description of the intervention

Once the teams have shown interest in collaborating in the study, the participating teams will be randomly assigned by clusters to the intervention or control group, as specified in the methodology.

There will be three visits (at the start of the study, at three and six months after inclusion in the study for each baby included in the study (control and intervention group). These visits will be used to collect necessary information.

The teams included in the intervention group will be given a two-hour specific training on passive smoking, epidemiology, morbimortality and how to intervene in the reduction of ETS. The proposed intervention is based on interventions recommended in primary care that follow international and national clinical practice guidelines [13,35,36]. The intervention is based on counseling, cognitive theory and motivational interviewing.

Thus, this study is proposing a passive smoking intervention that has been adapted from an intervention that has been shown to be effective in decreasing active tobacco consumption in Primary Care.

The intervention aims to give parents that smoke brief information that emphasizes the health benefits that reducing ETS can have on their children. The basic elements of the intervention include:

1. Ask: whether or not there is a smoker that lives with the baby. If the answer is affirmative, the baby's level of exposure will be asked.

2. Assess knowledge: Information that the parents have about the effect of passive smoking in infancy.

3. Personalized information depending on the exposure and the perceived risk.

4. Ask about suggestions to make changes: support any efforts to modify exposure to ETS and discuss the problems this entails.

5. Personalized help and advice according to exposure, baby's background, perceived risk and parents' attitude towards making changes. This intervention will be reinforced with educational leaflets about passive smoking in infancy designed for the study (Additional file 3).

6. Congratulate if the mother and/or father has quit smoking during the pregnancy and support-encourage continued smoking cessation.

7. Support and encourage those that decide to quit smoking or reduce tobacco consumption.

The control group teams will conduct the three proposed visits during the six-month follow-up period. Aside from collecting the study variables, the control group will carry out the protocol's habitual care for a healthy child during the corresponding visits. In Catalonia, these visits are officially scheduled and include a generic reference to avoid exposure to air polluted by cigarette smoke; however, there is no specific protocol for action.

### Database

The database will be obtained from medical records, questionnaires (interviews or visits), biological hair samples from the babies in the first visit and from expired carbon monoxide (CO) of parents that claim to have quit smoking.

### Follow-up period

The follow-up period is six months from the start of the intervention.

A visit will be conducted by the pediatrician/nurse at three and six months after the start of the study. They will register the parents' tobacco habit and the level of ETS exposure obtained by a questionnaire (at three and six months) and the nicotine in hair (at six months).

### Statistical analysis

Statisticians from the University of Girona's Research Group in Statistics, Applied Economics and Health (GRECS) will carry out the statistical analyses. These statisticians will have an advisory role.

The data will be incorporated into a database constructed using SPSS version 15.0. The descriptive analyses, as well as the statistical hypothesis testing, will also be conducted with this program. For subsequent modeling, the Free R software (version 11.0 or later) will be used.

All of the known potential confounding factors will be measured at the beginning of the study, including the caretaker who smokes (and the stage of change), level of education, marital status, breastfeeding and other members of the household, overcrowding. Therefore, the control and intervention group comparisons will be carried out both adjusted and unadjusted for these known confounders.

The simple treatment effect without adjusting the rates, relative risks and the 95% confidence intervals will be obtained in the first case, with a subsequent adjusted multiple regression analysis for other variables. Two forms of regression analysis will be taken into account for the primaries.

Expected results: A Poisson regression analysis and a negative binomial if there is evidence of over or under dispersion. The analysis of the secondary results will be carried out using standard statistical procedures applicable to categorical or continuous data. An adjustment for protocol analysis will also be conducted to verify the robustness of the results. All tests of significance will be two-tailed. For the treatment effects, sensitivity analyses will be conducted to determine the effect of the missing data.

The analysis will be done following the following study phases:

1. Describe the effect in both groups:

Initially an analysis of baseline comparability will be carried out in relation with the study variables. The following step will be to estimate the effect on the response variables without adjusting for other variables. To perform the above analyses, chi-squared tests will be used for qualitative variables and comparison of means for quantitative variables.

2. To estimate the adjusted effect, a multivariate multilevel model will be used. The dependent or principal variable is the child's exposure using the measure of nicotine in hair. The baby's age and sex, as well as the variables that have been shown to be statistically significant, will be included in the bivariate analyses. The basic care unit and the individual will be the levels in the multilevel analysis.

3. Concordance between the filled questionnaire and the nicotine collected in the hair: a summary evaluation of the questionnaire on a discrete, quantitative scale based on the data that is provided by the number of smoking cohabitants, the number of cigarettes that they smoke and the exposure time outside of the home. Pearson's (or Spearman's depending on the distribution) correlation coefficient will be calculated between the estimate and the result obtained in the nicotine hair analysis. The intraclass correlation coefficient will also be studied between both measures. Finally, both estimates will be dichotomized (questionnaire and nicotine in hair analysis) into two categories (exposed/not exposed) and the Kappa coefficient between the two will be calculated.

In all cases a bilateral alpha error of 0.05 and a 95% confidence interval will be used. To complete the explicit information, the comparison of means will be performed using a Student's t-test for parametric variables and the U Mann-Whitney test for non-parametric variables.

## Results

Primary results to obtain:

Reduction of exposure to ETS measured at the beginning of the intervention (recruitment phase) and at six months, using a questionnaire that includes information about ETS history during pregnancy and breastfeeding, exposure to ETS within and outside of the home, family smoking habits and measures used to avoid exposure. To develop this questionnaire, questionnaires used in similar studies have been adapted (varying some items, based on the results of the pilot study). As an objective measurement of the exposure: biological levels of nicotine in hair of babies. All of the hair samples (from the pilot test and the definitive study) will be analyzed by the same team at IMIM.

Secondary results to obtain:

- Reduction or cessation of smoking habit in parents, measured using a questionnaire about status of the smoker/ex-smoker/non-smoker and using the Fagerström Nicotine Dependence Test in those that smoke, as well as the stage of change (pre-contemplation, contemplation, preparation, action, maintenance, finalization). In smokers that declare smoking abstinence, the amount of carbon monoxide in expired air will be determined (Smokerlyzer). Cut-off point: 10 ppm.

-Other variables using questionnaire/clinical records, including data related to:

Type of professional that performs the intervention: pediatrician, pediatric nurse or both.

The baby: age, sex, breastfeeding, medical history, living nucleus.

The father and/or mother that attends the visit: age, sex, marital status, socioeconomic situation.

Parents' level of knowledge and their level of risk perception to exposure.

### Quality control

Several procedures have been employed to ensure the quality of the study data, maximizing the validity and reliability of the data obtained and of the evaluation of the results: These are:

-Written documentation: electronic and printed copies of protocols, models and outlines of informed consent stored together. All written documentation, including the leaflets given to participants, have been created according to a standard model and have been subject to the approval of local institutional ethics committees.

-A pilot study has been conducted to reveal deficiencies in the study design.

-An informational meeting will be held for both the intervention and control group to distribute documentation and to explain and materials.

-Learning: All of the intervention group professionals will receive the same two-hour workshop on passive smoking, attributable morbimortality and how to reduce exposure to ETS.

-Meetings will be held and regular emails will be sent regularly between the members that lead the study and all of the participants from the centers.

### Ethical aspects

The study and its successive revisions are adapted to the criteria of the Helsinki Declaration, as well as to the Guidelines to Good Clinical Practice. The protocol has been reviewed and approved by the CEIC" Clinical Research Ethics Committee" of the Jordi Gol and Gurina Foundation for the Promotion of Research in Primary Care (IDIAP Jordi Gol), with the number P08/50 "BIBE".

In regards to parents' informed consent, the information is provided verbally and written to all participants. Individuals in the study will have the opportunity to resolve doubts about study details. The written consent states that the study follows the law contained in the Helsinki Declaration and in Title I, Article 12 of the Royal Spanish Decree 561/1993 from April 16^th^, 1993.

Data confidentiality: Participants will be informed that data will be treated with absolute confidentiality according to the organic law that regulates the confidentiality of computerized data (Organic law 5/1992), and that data will be used exclusively for the objectives of the study.

Voluntary withdrawal: A mother or father can voluntarily withdraw his or her baby from the project at any point without having to give an explanation. He or she also has the right to ask that their personal data be eliminated from the records at any point, as well as for the results of their interviews.

### Limitations

The following limitations have been taken into consideration:

Professionals, rather than subjects (babies) will be randomized. Although this reduces the variability of the sample studied, it is necessary in order to minimize the possibility of contamination between groups.

The intervention will be carried out only in parents that visit the centers that are being studied, thus excluding a group of people that use other care services or that do not attend scheduled appointments. This phenomenon is present in normal clinical conditions, and the research team wants to show utility and feasibility in normal conditions, such as the ones taken into consideration in this design.

There could be a contamination effect in the control group due to the pre-test and the feeling of being observed during the study.

Intervention to prevent exposure to ETS will be carried out on the person who attends to the primary care consultation, therefore it can influence smoking status or not. For instance, the response to the intervention to prevent ETS exposure to babies could be greater or lesser according to the smoking status of the person. To estimate the possible effect of this phenomenon we will collect the status of tobacco consumption of the caretaker as well as of the other persons living with the child.

## Discussion

Exposure to ETS is an avoidable factor related to infant morbimortality. Interventions to reduce exposure to ETS in babies are potentially beneficial for their health.

As discussed in the introduction, increased exposure is declared in places not included in the Spanish tobacco law 28/2005. This justifies the need to define and promote a pediatric program in Primary Care that includes awareness interventions in the family environment, in places not included in the law (bars and cafes) or places that cannot be regulated because they belong to the private sphere (homes and cars), in order to achieve processes of change and protect infants.

The BIBE study evaluates, in real conditions, an intervention to reduce exposure to ETS that takes advantage of pediatric visits. The intervention that is proposed in the BIBE study aims to impact the attitude and knowledge of parents regarding ETS. Interventions in the form of advice, conducted by pediatric professionals, are an excellent opportunity for prevention and protection of infants against the harmful effects of ETS. In our country, health centers have almost universal coverage, including all social classes and situations. In fact, if the study proves to be effective it would also be efficient, since the intervention uses the same professionals that are responsible for the routine care of newborns.

Based on the bibliography, more research in passive smoking that uses objective measures of evaluation is necessary. Such research will generate evidence on the information that we are interested in collecting in order to learn children's exposure to cigarette smoke. It will also help to identify which intervention is effective in reducing this exposure. This is what we aim to do in the BIBE study.

## Abbreviations

BIBE: Brief Intervention in babies. Effectiveness; PS: Passive smoking; ETS: Environmental tobacco smoke.

## Competing interests

The authors declare that they have no competing interests.

## Authors' contributions

GO was responsible for the conception and design of the study, conceived and participated in the design of the questionnaires, led the development of the program and the training and wrote the firsts drafts and final version of the study protocol. CC, CM, JB, ED, JL, LR, CM, CC, ES, AV, and MJ contributed to the design and development of the study and the questionnaires, as well as to the writing and development of the protocol. They have also contributed to the development of the program and the training. MS and MB were responsible for the statistical design of the protocol, the randomization and the statistical analysis of the pilot study data. JP and RP analyzed hair concentrations of nicotine.

The associated researchers contributed to the field work: recruitment, interview and collection of hair samples. Those in the intervention group have contributed to the development of the intervention protocol.

All authors have performed a critical revision of this manuscript and the final version.

## Pre-publication history

The pre-publication history for this paper can be accessed here:

http://www.biomedcentral.com/1471-2458/10/772/prepub

## Supplementary Material

Additional file 1 **Image 1. Cut a lock of the hair**. Images showing how to cut a lock of baby's hair. Click here for file

Additional file 2 **Image 2. Sample of the baby's hair to label and send**. Image of a sample of baby's hair that was used to analyze nicotine concentration. Click here for file

Additional file 3 **Image 3. Leaflet: Passive smoking: advice to decrease exposure in babies**. Image showing a leaflet in Catalan which contains correct and incorrect advices for parents to reduce baby's exposure to environmental tobacco smoke. Click here for file
