# Supplementary figures and images for: Passive smoking in babies: The BIBE study (Brief Intervention in babies. Effectiveness)
Source: BMC Public Health. 2010 Dec 20;10:772. doi: 10.1186/1471-2458-10-772 (PMC3019194; doi:10.1186/1471-2458-10-772)

Image 1. Cut a lock of the hair

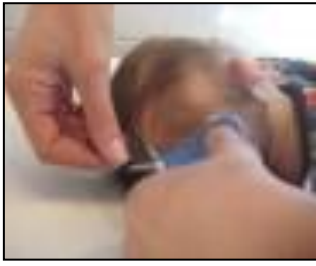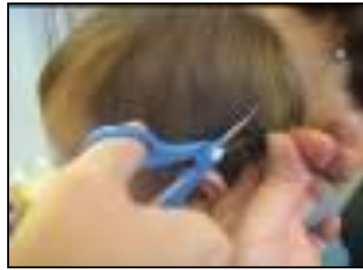

Supplement: Additional file 1 — Image 1. Cut a lock of the hair. Images showing how to cut a lock of baby's hair. [file 1471-2458-10-772-S1.PDF]

Image 2. Sample of the baby's hair to label and send

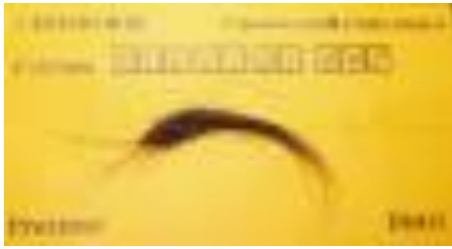

Supplement: Additional file 2 — Image 2. Sample of the baby's hair to label and send. Image of a sample of baby's hair that was used to analyze nicotine concentration. [file 1471-2458-10-772-S2.PDF]
