# Supplementary material for: Passive smoking in babies: The BIBE study (Brief Intervention in babies. Effectiveness)
Source: BMC Public Health. 2010 Dec 20;10:772. doi: 10.1186/1471-2458-10-772 (PMC3019194; doi:10.1186/1471-2458-10-772)

Image 3. Leaflet: Passive smoking: advice to decrease exposure in babies

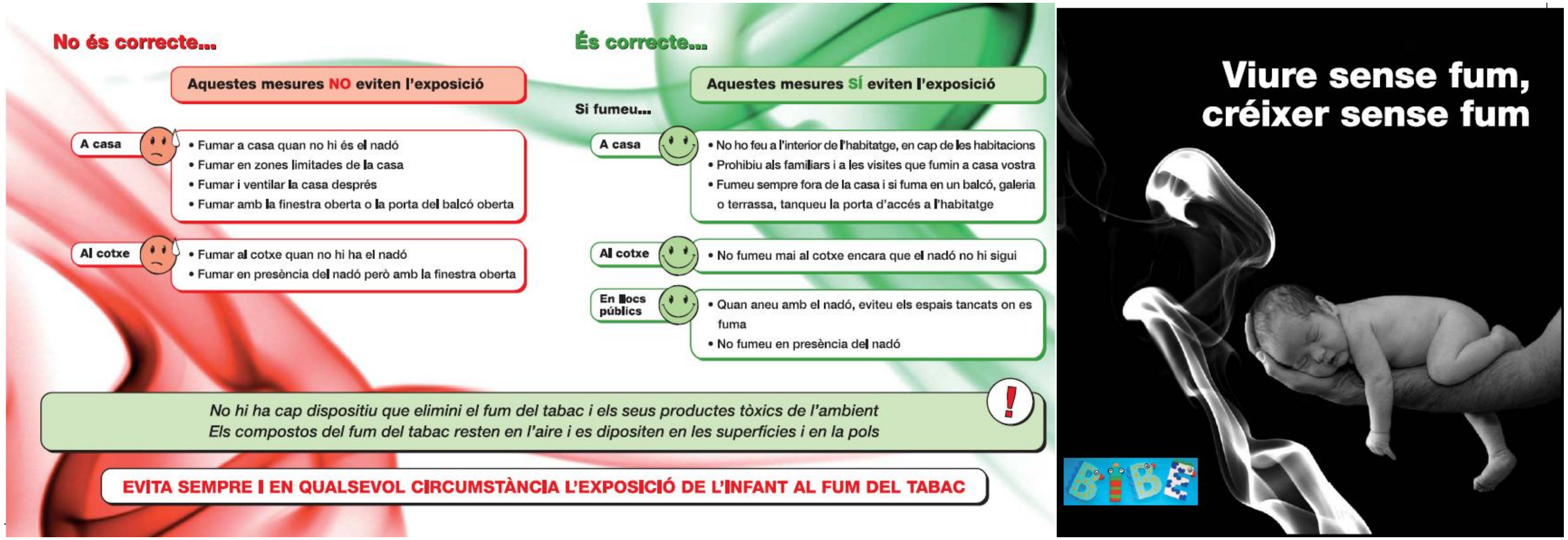

Supplement: Additional file 3 — Image 3. Leaflet: Passive smoking: advice to decrease exposure in babies. Image showing a leaflet in Catalan which contains correct and incorrect advices for parents to reduce baby's exposure to environmental tobacco smoke. [file 1471-2458-10-772-S3.PDF]
